# Using Ecological Null Models to Assess the Potential for Marine Protected Area Networks to Protect Biodiversity

**DOI:** 10.1371/journal.pone.0008895

**Published:** 2010-01-27

**Authors:** Brice X. Semmens, Peter J. Auster, Michelle J. Paddack

**Affiliations:** 1 Northwest Fisheries Science Center, Seattle, Washington, United States of America; 2 Department of Marine Sciences and National Undersea Research Center, University of Connecticut, Groton, Connecticut, United States of America; 3 Department of Biology, Santa Barbara City College, Santa Barbara, California, United States of America; University of California San Diego, United States of America

## Abstract

Marine protected area (MPA) networks have been proposed as a principal method for conserving biological diversity, yet patterns of diversity may ultimately complicate or compromise the development of such networks. We show how a series of ecological null models can be applied to assemblage data across sites in order to identify non-random biological patterns likely to influence the effectiveness of MPA network design. We use fish census data from Caribbean fore-reefs as a test system and demonstrate that: 1) site assemblages were nested, such that species found on sites with relatively few species were subsets of those found on sites with relatively many species, 2) species co-occurred across sites more than expected by chance once species-habitat associations were accounted for, and 3) guilds were most evenly represented at the richest sites and richness among all guilds was correlated (i.e., species and trophic diversity were closely linked). These results suggest that the emerging Caribbean marine protected area network will likely be successful at protecting regional diversity even if planning is largely constrained by insular, inventory-based design efforts. By recasting ecological null models as tests of assemblage patterns likely to influence management action, we demonstrate how these classic tools of ecological theory can be brought to bear in applied conservation problems.

## Introduction

Loss of biodiversity is a critical ecological and conservation issue [1], [2]. Evidence of increasing threats and drastic declines in marine ecosystems is mounting [3], [4], [5], [6]. Marine protected areas (MPAs) and MPA networks have become a fundamental tool in conservation planning because they have the potential to address a broad array of management goals, including but not limited to biodiversity conservation [7], [8]. The extent to which MPA networks protect biodiversity depends in part on the selection of sites that maximize the representation of biodiversity. Several systematic conservation planning strategies have been created in order to identify reserve networks that address such specific conservation goals [9], [10], [11], [12]. However, even with effective planning strategies, the establishment of MPA networks is often constrained by political, financial, and informational limitations; these limitations may result in a realized MPA network that falls far short of the optimal design [13], [14]. To what extent are the diversity conservation goals of such MPA networks compromised? The answer to this question will undoubtedly depend on the details of MPA configuration, size and placement, as well as the characteristics of connectivity among sites. However, studying patterns in diversity and community composition across sites can help identify general characteristics of assemblages likely to either hinder or facilitate effective reserve design.

Efforts to conserve regional-scale diversity in MPA networks may be buffered or confounded by non-random patterns in species assemblages. A systematic evaluation of assembly patterns can yield insights into the extent to which such patterns (and their underlying processes) are likely to influence effective network design. For instance, MPAs are typically established through insular planning efforts [e.g., 15] that emphasize local but not regional patterns of diversity. The extent to which MPA networks based primarily on α- (local) diversity can adequately protect γ- (regional) diversity depends at least in part on how species are distributed across sites and in relation to other species. In fact, even the goal of protecting α-diversity through MPA networks may be difficult to achieve if different forms of diversity (e.g., species vs. trophic) are relatively unrelated and thus difficult to account for simultaneously [16]. Clearly, the extent to which a MPA network can adequately conserve diversity across a region depends in part on assemblage patterns across potential MPA sites.

Ecological null models offer a framework for examining patterns in species distributions given the large body of literature supporting their theoretical and statistical underpinnings. Inferring ecological processes in community assembly based on patterns in the presence or absence of species across sites has been a central line of inquiry in the ecological literature [17], [18], [19]. The utility of these tests has been criticised because the specific biological processes responsible for the observed patterns can be difficult to tease apart [19]. However, in a conservation context the presence or absence of patterns can be highly informative even if the underlying processes are not evident.

In this paper we recast a series of classic ecological null models as tests for patterns in fish assemblages likely to compromise efforts at conserving regional reef fish diversity in Caribbean MPAs. These tests are based on patterns of nestedness [20], guild proportionality [21], and species co-occurrence [17]. To draw inferences regarding how the patterns revealed by these tests might influence diversity-based MPA network design efforts, we focus on the planning goal of maximizing the number of species included in a MPA network. This is a commonly used MPA planning goal in both theory and practice [15], [22], [23]. We used the Caribbean region as a case study due to the availability of spatially coherent region-wide data and a growing interest in establishing MPA networks across national and territorial jurisdictions [24]. While our analyses are limited to only one aspect of biodiversity (forereef fishes) and produce results largely specific to the Caribbean, our intent is to demonstrate how these classic tools of ecological theory can be brought to bear in applied conservation problems.

## Methods

**Figure 1:**
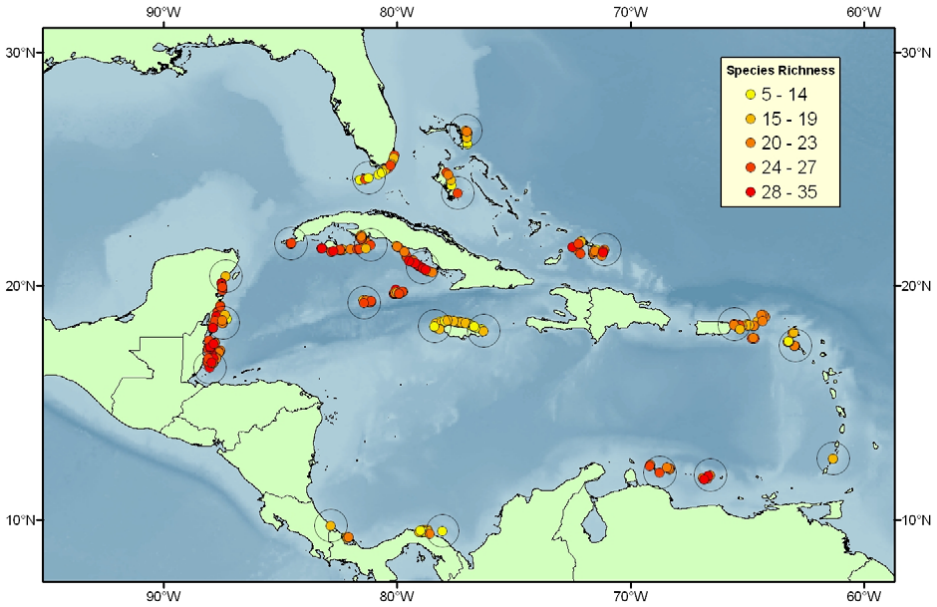
The geographic distribution of survey sites used in our analysis of reef fish diversity in the Caribbean basin. The sites circled by black rings represent the 20 (∼5% of all sites) most geographically separate sites based on a maximization of the minimum Euclidean distance between all sites.

All analyses were based on a presence-absence matrix of 63 species (Table S1) from 373 Caribbean forereef sites surveyed as part of the Atlantic and Gulf Reef Rapid Assessment (AGRRA; [25] from 1997 through 2003 (Figure 1). The AGRRA program was developed to assess western Atlantic reef communities with a standardized method. Researchers participating in AGRRA learned survey techniques through standardized training programs. The AGRRA methodology recommended 10 fish surveys (2 m×30 m transects) per site, which was achieved at most sites. We excluded sites outside the Caribbean basin and those sites that lacked data for any of the 14 site characteristics required for the species co-occurrence null model (described below). We used only forereef sites to minimize variability in assemblages due to associations between species and geomorphologic reef zones [26], [27]. Finally, we classified the protected status and Caribbean ecoregion of sites using the World Database on Protected Areas [28] and the Marine Ecosystems of the World database [29], respectively. Following the IUCN definition of a protected area, we characterized sites as protected if they were located in an area “dedicated to the protection and maintenance of biological diversity, and of natural and associated cultural resources, and managed through legal or other effective means”. Seven different ecoregions were represented by the sites included in the analysis: Bahamian, Eastern Caribbean, Floridian, Greater Antilles, Southern Caribbean, Southwestern Caribbean, and Western Caribbean.

The AGRRA protocol surveys all members from 6 families of fish (Acanthuridae, Chaetodontidae, Haemulidae, Lutjanidae, Scaridae, and Serranidae) and selected members from 7 families (Balistidae, Carangidae, Sphyraenidae, Labridae, Monacanthidae, Pomacanthidae and Pomacentridae; Table S1). Following Bellwood and Hughes (2001 [30]), we relate our findings to total reef fish diversity based on the fact that diversity patterns are highly correlated among families [31].

Because any future efforts to establish an MPA network in the Caribbean would build upon existing MPAs, we first evaluated the extent to which existing MPAs incorporate regional diversity. We approached this evaluation by addressing the question “does the current MPA network tend to protect high diversity sites?” To do this analytically, we fit a generalized linear mixed model with site-specific reef fish richness as the response (modelled as Poisson), a fixed effect term for the protection status of sites, and random effects for the ecoregions. We included ecoregion random effects to avoid psuedoreplication due to spatial autocorrelation in assemblages [29].

Below we introduce each of the ecological null model tests employed by 1) describing the implications of the test results for MPA network design, and 2) outlining the specific analytic methods used to carry out the tests. All tests required the development of null matrices through constrained randomizations of the species by site matrix. Type I error probabilities were evaluated by comparing the test statistic of the observed data with the distribution of test statistics from 10,000 null matrices. We calculated one-tailed probabilities as: (number of null test statistics equal to or larger than the observed test statistic+1)/(number of randomizations+1).

### Nestedness

In a nested system, rare species occur at relatively diverse sites more frequently than expected by chance [20]. This may not be the case if, for instance, rare species tend to specialize on resource sets unique to species-poor sites. Ecological processes that may yield nested assemblages include variability in extinction probabilities, differences in colonization rates, or an underlying pattern of habitat nestedness [32]. Developing a MPA network in a nested system by focusing on α-diversity is likely to coincidentally conserve γ-diversity. That is, local governments that act to protect their most diverse sites in a nested system will coincidentally tend to optimize their contribution to a regional network.

We determined nestedness by calculating the matrix ‘temperature’ [20]. The matrix temperature measures the degree to which the species incidences in a species by site matrix depart from perfect nestedness (when sites are ordered from most to fewest species, each subsequent site in the order will have a proper subset of species in the previous site). Lower temperatures connote higher nestedness. We determined the Type I error probability by comparing the temperature of the data matrix against temperatures calculated from null matrices. In order to avoid artificially lowering the Type I error probability due to passive sampling of skewed species abundance distributions [33], we generated constrained null matrices such that the proportional occurrence of each species was always exactly equal to the original data matrix.

### Species Co-Occurrence

A large body of theoretical work has focused on methods for detecting non-random co-occurrence patterns in species-by-site presence-absence data [18]. In reef fish assemblages, competition may [34] or may not [35] limit species co-occurrence (and by implication diversity), whereas predation may exert a negative [36] or positive force [37] on co-occurrence. Indirect effects [38] and facilitation [39] may similarly influence co-occurrence. Patterns in species co-occurrence can also reflect factors such as variable anthropogenic impacts across sites (e.g., selective fishing pressure), or habitat heterogeneity and species specific habitat affinities [40], [41]. To some extent the influence of factors other than species interactions can be factored out of null model analyses by generating null matrices that account for these other factors [42].

Methods for controlling the influence of habitat heterogeneity and anthropogenic impacts on reef fish assemblages allowed us to evaluate species co-occurrence patterns when MPA sites are chosen in order to protect representative habitats and a variety of human uses [e.g., 43]. If species co-occur less than expected by chance even after accounting for habitat heterogeneity across sites, then the number of MPA sites required to protect a given number of species will coincidentally be higher than expected. Similarly, fewer MPAs will be required to protect a given number of species in a system with higher species co-occurrence.

If species tend not to co-occur, the species by site matrix will exhibit a high degree of ‘checkerboarding’ (i.e., when species A is present, species B is absent, and vice versa) when compared with null community matrices [c-score test, 44]). Conversely, if species are positively associated, species will tend to exhibit a high degree of ‘togetherness’ (i.e., species A and B are jointly either present or absent) when compared with the null community matrices [t-score test, 44]). We used two methods to develop constrained null matrices in order to evaluate the Type I error probabilities of the c- and t-scores of the data matrix. First, we generated constrained null community matrices using a swap algorithm [40] where the number of occurrences of each species across all sites and the number of species at each site is set exactly equal to the original data matrix; null matrices were not limited in terms of the ability of members of the same guild to co-occur. Second, because the swap algorithm does not explicitly account for site-specific habitat characteristics, we conducted a separate test using methods described in Peres-Neto et al. (2001 [42]) to generate null community matrices based on site-specific species' occurrence probabilities derived from habitat suitability analyses [Table S2, 42]). Null assemblages for each site were therefore based on species-specific relationships between both local (e.g., depth, benthic cover, rugosity, wave exposure, fishing pressure) and regional (e.g., degree of isolation from other reef areas, location along latitudinal and longitudinal gradients) site characteristics. This latter approach allowed us to evaluate how co-occurrence might influence a MPA network implemented in order to maximize representative habitats and human uses.

We determined site suitability by conducting a discriminant analysis for each species using presence/absence data across sites as the response parameter, and 14 site-specific habitat characteristics (including an index of fishing pressure; Table S2) as predictor variables. Using all site characteristics, the discriminant functions on average correctly predicted presence-absence of species across sites 74.5% of the time. Peres-Neto et al. (2001 [42]) describe two algorithms for developing null communities: 1) Ct-RA1, which exclusively uses presence/absence probabilities derived from discriminant functions (used when these functions have good predictive power), and, more conservatively, 2) Ct-RA2, which adjusts presence/absence probabilities for each site based on the actual presence/absence of a species (used when discriminant functions have poor predictive power). Regardless of which algorithm we used, our results remained unchanged.

### Guild Proportionality

The guild proportionality null model [21] tests the probability that the variance in guild proportions across sites is lower than expected by chance. In this paper we applied the guild proportionality test to reef fish feeding guilds. Trophic diversity plays an important role in reef health and function, and should be accounted for in MPA site selection [45], [46]. For example, reefs supporting herbivores with a variety of morphological and behavioural feeding mechanisms will be more resilient to perturbation [47]. Ideally, trophic diversity occurs proportionally across sites regardless of species richness. In such a scenario, diversity within trophic groups would be monotonically related to species diversity such that the sites with the most total species would tend to also have the most species from each feeding guild.

To evaluate guild proportionality we first grouped species into mutually exclusive trophic guild classifications so that each species represented only one guild (Table S1; [48]). Next, we generated null matrices with the occurrences of each species across all sites and the number of species at each site set exactly equal to the original data matrix. We subsequently compared the variance of guild proportions in the original data matrix to the distribution of variances from the null matrices. Decreasing the number of trophic categories to the point where we could place species into exclusive trophic guilds, while necessary for the null model test, sacrificed a considerable amount of detail regarding the species' feeding ecology. A finding of statistical support for guild proportionality when using relatively few trophic categories would not necessarily imply that proportionality holds at greater trophic resolutions. Nonetheless, guild proportionality, even with coarse trophic categories, would provide evidence that at least some forms of diversity are coupled more than expected by chance.

## Results

Reef fish diversity at sites inside existing protected areas (n = 139) was on average 15% higher than at unprotected sites (n = 233, p<0.0001; Table 1). Additionally, 17 of the 20 richest sites (∼5% of all sites) were located inside existing protected areas.

**Table 1:** Results from a generalized linear mixed model relating richness at 373 sites (number of species observed, Poisson response) to the reserve status of sites (reserved or not, fixed effect) and the ecoregion of sites (random effects).

Random effects				
Groups	Num. Groups	Variance	Std. Dev.	
Ecoregions	7	0.021	0.142	

Fixed Effects				
	Estimate	Std. Error	z value	Pr(>|z|)
Intercept	2.971	0.056	53.58	<2e-16
Reserved	0.128	0.024	5.32	1.03e-07

**Figure 2:**
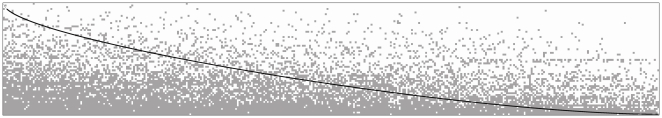
Maximally packed presence-absence matrix of Caribbean reef fishes, such that species are sorted in order of ubiquity (rows) and sites are sorted in order of richness (columns). Each darkened square indicates the presence of a species at a site. The curve represents the isocline of perfect nestedness subject to the constraints of perfect occupancy at the most diverse site, and perfect vacancy at the least diverse site.

**Figure 3:**
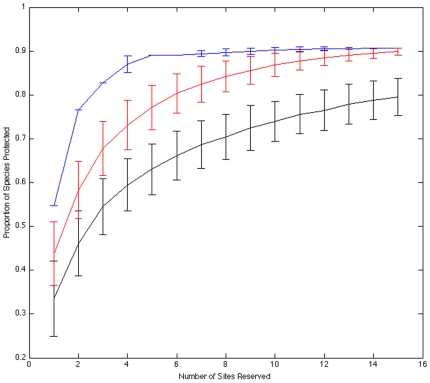
Reserve network performance under three selection scenarios: 1) sites selected at random (black line), 2) sites selected by choosing the single richest site from each jurisdiction (red line), and 3) sites selected by choosing the richest sites regardless of jurisdiction (blue line). We used the 15 country designations specified in the AGRRA database as our jurisdictional units. Each line represents the proportion of all species protected as a function of the number of reserved sites under each of the three scenarios. Error bars are ±1 standard deviation. For scenarios 2 and 3, when two or more sites had the same richness, one of these sites was selected at random (thus, scenario 3 has error bars). Similarly, when selecting fewer sites than the total number of jurisdictions considered in scenario 2, we randomly identified jurisdictions to choose sites from (thus, error bars decrease as the number of reserves approaches the number of jurisdictions).

Caribbean reef fish assemblages were highly nested across sites (p<0.0001; Figure 2). While on average each site contained 30% of the total species pool, the 15 richest sites represented ∼90% of that pool (Figure 3). Given that the richest sites are unevenly distributed throughout the Caribbean (Figure 1), some governments have the potential to contribute more to the protection of regional diversity than others. However, because assemblages are highly nested, any government that acts to protect diversity within their jurisdiction will coincidentally maximize their potential contribution to a regional MPA network regardless of past or future actions by other governments. For instance, selecting the single richest site within each of the 15 country designations represented in our data set culminated in the preservation of approximately the same proportion of species as a MPA network composed of the richest 15 sites regardless of jurisdiction (Figure 3).

Without explicitly accounting for differences in habitat and fishing pressure across sites, reef fish assemblages exhibited a high degree of checkerboarding (c-score test, p<0.0001; t-score test, p>0.9999). However, when we explicitly accounted for differences in habitat suitability across sites, we obtained the opposite result: reef fish assemblages exhibited a high degree of positive species co-occurrence (c-score, p>0.9999; t-score, p<0.0001). True reef fish richness correlated strongly with predicted richness using habitat suitability estimated from all 14 site characteristics (0.8788; p<0.0001). Nevertheless, residuals from the relationship were spatially auto-correlated (Figure S1), perhaps due to the clumped nature of sites (Figure 1). To investigate the influence of spatial autocorrelation on our findings, we repeated the c-score and t-score analyses on the 20 most geographically separate sites in the data set (∼5% of all sites). While the residuals from the relationship between true richness and predicted richness based on habitat suitability in these sites were not spatially auto-correlated (Figure S1), the co-occurrence test results remained the same.

**Figure 4:**
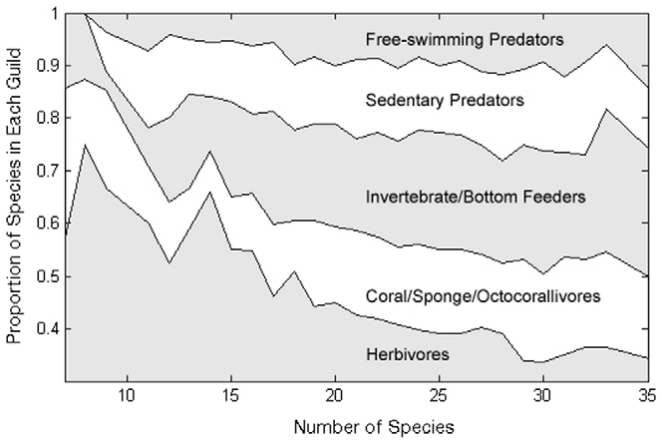
The average relative proportion (y-axis) of Caribbean reef fish assemblages represented by each trophic guild as a function of site richness (x-axis). Note that both predator trophic groups were completely absent from sites with relatively few species, suggesting that different forms of diversity may be decoupled at species poor sites.

The total site richness represented by each guild did not exhibit lower variance than expected by chance (p = 0.45), probably due to the predominance of the herbivore guild on species poor sites (Figure 4). On the other hand, the contribution of guilds to total site richness tended towards parity among guilds as site richness increased (Figure 4), and richness correlated positively for all pair-wise comparisons of guilds (Table 2).

**Table 2:** Pearson's correlation coefficients of reef fish guild diversity across Caribbean reef sites.

Guild	By Guild	Correlation	P-Value
Free-swimming predators	Sedentary predators	0.29	<0.002
Free-swimming predators	Invertebrate/bottom feeders	0.41	<0.002
Free-swimming predators	Coral/sponge/octocorallivores	0.25	<0.002
Free-swimming predators	Herbivores	0.20	<0.002
Sedentary predators	Invertebrate/bottom feeders	0.32	<0.002
Sedentary predators	Coral/Sponge/octocorallivores	0.44	<0.002
Sedentary predators	Herbivores	0.22	<0.002
Invertebrate/bottom feeders	Coral/sponge/octocorallivores	0.41	<0.002
Invertebrate/bottom feeders	Herbivore	0.22	<0.002
Coral/sponge/octocorallivores	Herbivore	0.11	0.04

P-values are adjusted for multiple hypothesis tests using the Holm-Bonferroni method [75].

## Discussion

To date, the establishment of MPAs has rarely proceeded with adequate information, raising concerns that emerging networks will perform poorly [45]. Given that MPA networks interact across jurisdictional boundaries and are necessarily built upon a foundation of existing MPAs, the addition of sites to a network typically proceeds in what is essentially an *ad hoc* manner. This is particularly true in the Caribbean basin, where a geo-politically complex and largely uncoordinated process drives the emerging MPA network. Fortunately, our study suggests that this emerging *ad hoc* Caribbean reserve network has done and will continue to do surprisingly well at conserving the γ-diversity of Caribbean forereef fishes. We caution, however, that such results are not necessarily transferable to other taxa or regions as assemblage patterns and the processes that drive them will vary across systems [49]. The important result here is that ecological null model analyses provide a powerful toolset for identifying patterns in assemblages that may either complicate or facilitate efforts aimed at conserving regional biodiversity. Because uncertainty regarding the future success of MPA networks is often cited as a reason to downsize or abandon the planning process, we expect that analytic tools that provide insight into the future performance of MPA networks will facilitate the planning process.

Describing species distribution patterns and their underlying processes is a fundamental part of ecological studies. In this study we used a suite of analytic methods aimed at isolating distribution patterns in order to infer the mechanisms driving the patterns. These inferential approaches are both necessary and appropriate at scales that are not experimentally tractable. However, even well designed null model tests yield patterns that could be explained by multiple processes–this has been a central criticism of the community assembly null model approach [50]. Our focus on the conservation implications of the patterns themselves (rather than the processes driving them per se) largely skirts this criticism; nonetheless, the mechanistic implications of the patterns identified warrant brief discussion before delving into the conservation and management implications of the species distribution patterns.

### Nestedness

Patterns in community nestedness likely reflect the cumulative influence of evolutionary history, the geographic isolation of sites, and habitat affinities among species. Identifying the specific ecological mechanisms behind nested patterns has proven challenging [51]. Many studies have identified habitat characteristics as an organizing factor in nested patterns [e.g.], [32], [50,52]; the strong association between species occurrence and habitat features identified in the application of the habitat-mediated co-occurrence null model analysis suggests that habitat plays an important role in structuring Caribbean reef fish assemblages, likely including the nested patterns identified in this study.

A MPA siting process typically accounts for diversity by balancing the goal of preserving α-diversity with the local socioeconomic impact of management actions. For instance, the diversity and habitat criteria used to evaluate siting alternatives for the Tortugas Ecological Reserve (Florida Keys, FL, USA) was “to choose an area that would contain the greatest level of biological diversity and that would encompass a wide range of different contiguous habitats” [15]. Given our finding that species assemblages within a habitat (forereef sites) and across jurisdictions are nested, it is likely that this ongoing insular approach to MPA network design (targeting α-diversity) will continue to do well at protecting γ-diversity. However, the habitat-mediated co-occurrence patterns revealed here highlight the importance of incorporating representative habitats as a design goal.

### Species Co-Occurrence

Positive species interactions in reef fish have been documented through both observational and experimental studies [e.g.], [social foraging], [synergistic predation, settlement facilitation, 53,54,55]), but to our knowledge this study is the first to present evidence that facilitation acts broadly to structure species distribution patterns among reef sites. This “diffuse” facilitation may provide a mechanistic explanation for the tendency of marine reserves to result in higher diversity following implementation [e.g., 56]). In other words, while it is easy to infer that anthropogenic pressures such as fishing are lowering diversity levels outside Caribbean MPAs, it may be the case that positive species interactions inside MPAs are simultaneously acting to increase diversity.

Efforts to establish MPA networks in order to conserve diversity often rely on species inventories, and presume that these inventories will remain the same following protections. Such a decision-making process implicitly assumes that communities are essentially constructs of species autecology. In contrast, a large body of research has focused on demonstrating that communities are constructs of ecological processes (e.g., [38], [36]). This latter form of community organization may complicate diversity-based MPA design efforts made without knowledge of or consideration for mechanisms underlying community composition. Undeniably, there are strong interactive and ecological forces at play within reef fish assemblages [57] but do these forces mediate species coexistence at the scale of MPAs in the Caribbean? The finding of positive species associations after accounting for habitat and human use differences across sites appears to rule out negative interactions as a major structuring force in the presence or absence of species across reef sites. If negative interactions do yield species ‘checkerboarding’, these effects are apparently swamped at the scale of our study by other factors such as nested habitat affinities beyond the site characteristics we controlled for in our co-occurrence null model. Regardless of the mechanistic explanation for positive species associations, their manifestation in community assembly implies that Caribbean MPA design efforts based on species inventories and representative habitats will likely be uncompromised by species interactions.

### Guild Proportionality

Diversity within functional groups (e.g., trophic guilds) is directly related to coral reef resilience—that is, the ability of a reef to regenerate from disturbance pulses, absorb disturbance presses, and resist phase shifts [58]. However, Caribbean coral reefs (as compared to tropical Pacific reefs) have relatively low diversity and redundancy in the regional species pool [45], [46]. This may in part explain the tendency for low diversity sites to have complete trophic “drop outs” of predator guilds (Figure 4). The link between predators and ecosystem resilience is non-trivial. Declines in predators have resulted in decreases in important primary producers within terrestrial and marine ecosystems [59], [60] and across ecosystem boundaries [61], leading to ecological degradation of these systems. On Caribbean coral reefs, large- bodied predators such as sharks and groupers appear to have particularly high interaction strengths, leading to higher potential for trophic cascades when they are removed [62]. Reductions in large-bodied predators may actually result in declines in recruitment of important grazing species (such as *Sparisoma viride*) due to increases in predation pressure from smaller-bodied predators [63]. Decreased abundance of such grazers has been linked to increases in macroalgal cover and consequent reduction in coral recruitment [64], [65]. Given the presumed importance of predator guilds to reef resilience, it is possible that low diversity sites may lack the functional diversity necessary to absorb anthropogenic impacts or recover from disturbance.

If differences in reef fish diversity across sites are driven primarily by changes in the richness of a subset of trophic groups, then targeting species-rich sites may not consistently protect trophic diversity. A disconnect between different forms of biological diversity would certainly complicate the MPA site selection process [45]. Our results suggest that while species-poor sites may lack a full complement of guild types [30], [66], a close linkage between species diversity and trophic diversity means that reasonable efforts by managers and MPA designers to include species-rich sites in MPA networks will *de facto* protect high trophic diversity sites.

### Caveats and Conclusions

The generality of our findings across larger spatial scales, different ocean regions, and different metrics of diversity should be tested rather than assumed. For instance, given endemism and the steep gradients in species richness across the tropical Pacific [30], it is unlikely that a MPA network developed by conserving the richest sites would approximate an optimal diversity-based network. Similarly, although the diversity of different reef-associated assemblages (e.g., fishes and corals) is often correlated, these correlations may be modest [30]. Forereefs are typically the most structurally and biologically complex reef areas, and consequently those of the highest species richness, particularly for fishes and corals [67], [68], [69]. Overall diversity of a reef system is therefore likely well captured by forereef diversity. Nonetheless, because our findings only apply to a specific geomorphic zone, generalization across zones requires the assumption that: 1) managers make similar efforts to represent habitats and species diversity across reef zones, and 2) the specific findings regarding community assembly are similar across reef zones. While these assumptions are reasonable [26], [27], it is possible they are inaccurate. Ideally future management applications of our approach will fully encapsulate the specific systems and habitats being considered for management action.

The link between site-specific habitat features and community assembly patterns identified in this study reinforces the notion that, in the absence of detailed spatially-explicit species richness data, habitat features may be effective proxies for species diversity [70]. On the other hand, the cumulative evidence that Caribbean reef fish assemblages are structured by ecological and evolutionary mechanisms suggests that failing to consider such mechanisms or their resultant species distribution patterns may yield MPA networks that fall far short of optimal network design.

The application and interpretation of multiple community assembly null model tests must be done with caution, as independence cannot necessarily be assumed. For example, nestedness and species co-occurrence are commonly applied in concert in studies of meta-community pattern, and it is clear that pairing these tests can improve understanding of non-random community organization. However, several researchers have noted that, at least superficially, these two tests seem to describe opposing community patterns—in a nested matrix, species tend to share sites, while species in a checkerboarded matrix tend to have limited overlap in occurrences [71], [72], [73]. Despite the apparent negative relationship between to the two metrics, researchers have reported matrices that are at once nested and checkerboarded. What, then, is the explicit relationship between the two metrics? Ulrich and Gotelli (2007 [71]) systematically explored the relationship using simulated matrices with varying degrees of nestedness, checkerboarding, and randomness. They found that for a given matrix configuration, nestedness and checkerboarding are loosely related (R^2^ = ∼0.30), but that the sign of the relationship (positive or negative) depended on the configuration and fill of the matrix. Urlich et al. (2009 [51]) found a similarly weak relationship between measures of co-occurrence and nestedness when null matrices were simulated under row and column sum constraints. Thus, matrices with negative species co-occurrence may or may not be nested depending on the dimensionality and percent fill of the matrix being tested. In the context of our study, these findings imply that the application of both tests is not duplicative, but rather a reasonably independent assessment of nested species subsets and species segregation across Caribbean reef sites.

Uncertainty, ecological surprises, and the increasing degradation of marine ecosystems, particularly tropical [4], [74], unquestionably warrant calls for active, systemic management of these ecosystems [45]. Putting such an approach into practice requires information sufficient to characterize important ecosystem roles and services, and a degree of governmental cooperation sufficient to implement conservation at an appropriate scale [8]. Our results suggest that the culmination of disjointed local efforts can do surprisingly well at conserving regional diversity in the Caribbean. The analytic methods we employed integrate across entire assemblages to evaluate pattern. While this is a common approach in macroecological studies, it may discount the details of natural history at the peril of successful conservation actions [16]. When knowledge of such details is scarce however, elucidating and interpreting diversity patterns across sites can help meet the information needs required to drive successful conservation actions.

## Supporting Information

Figure S1 Moran's I correlogram of residuals from the linear relationship between true site richness and predicted richness based on an analysis of habitat suitability for each species. Figure S1a presents findings from an analysis that included all survey sites, while figure S1b presents findings from an analysis of the 20 most geographically separate sites in our analysis. Positive values indicate positive spatial autocorrelation, and negative values indicate negative autocorrelation. Solid circles connote significant autocorrelation at the distance indicated (p<0.05) while open circles connote non-significance. Note that none of the Moran's I values from the analysis of the 20 most spatially separate sites were significant.
(5.43 MB BMP)

Table S1 Families, scientific names, common names, and exclusive trophic guild associations (derived from Randall 1967) of all fish species recorded from AGRRA sites used in this study.
(0.08 MB DOC)

Table S2 Habitat characteristics used to conduct the environmentally constrained null model co-occurrence tests, along with the methods used to quantify each characteristic for all sites. Citations given in the last column postulate, document through observation, or experimentally demonstrate associations (either positive or negative) between reef fish assemblage metrics (e.g., richness, recruitment, biomass, ordination) and the habitat characteristic.
(0.12 MB DOC)
