# Supplementary figures and images for: Using Ecological Null Models to Assess the Potential for Marine Protected Area Networks to Protect Biodiversity
Source: PLoS One. 2010 Jan 27;5(1):e8895. doi: 10.1371/journal.pone.0008895 (PMC2811735; doi:10.1371/journal.pone.0008895)

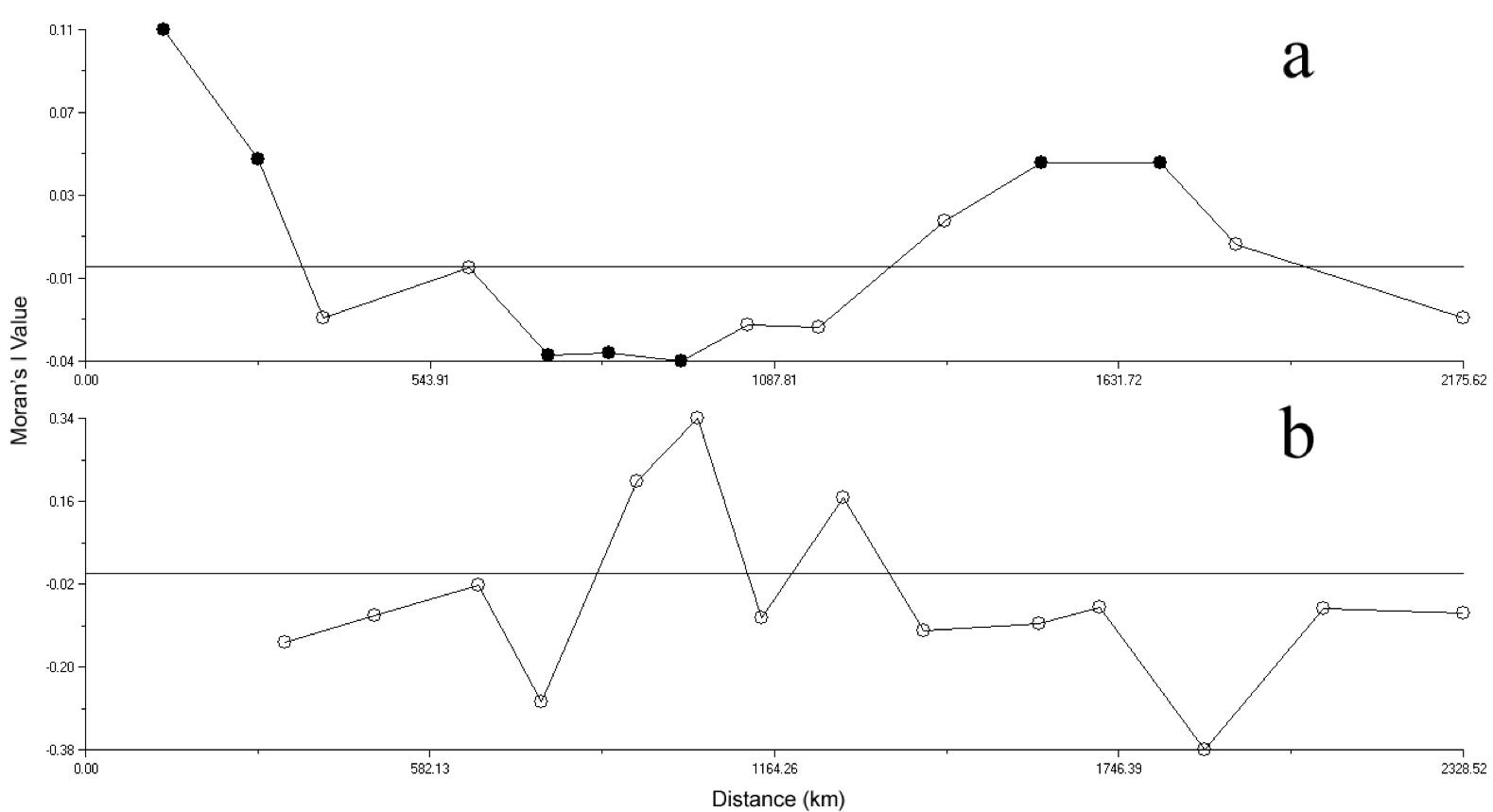

Supplement: Figure S1 — Moran's I correlogram of residuals from the linear relationship between true site richness and predicted richness based on an analysis of habitat suitability for each species. Figure S1a presents findings from an analysis that included all survey sites, while figure S1b presents findings from an analysis of the 20 most geographically separate sites in our analysis. Positive values indicate positive spatial autocorrelation, and negative values indicate negative autocorrelation. Solid circles connote significant autocorrelation at the distance indicated (p<0.05) while open circles connote non-significance. Note that none of the Moran's I values from the analysis of the 20 most spatially separate sites were significant. (5.43 MB BMP) [file pone.0008895.s001.bmp]
