# Supplementary material for: Using Ecological Null Models to Assess the Potential for Marine Protected Area Networks to Protect Biodiversity
Source: PLoS One. 2010 Jan 27;5(1):e8895. doi: 10.1371/journal.pone.0008895 (PMC2811735; doi:10.1371/journal.pone.0008895)
Supplement: Table S1 — Families, scientific names, common names, and exclusive trophic guild associations (derived from Randall 1967) of all fish species recorded from AGRRA sites used in this study. (0.08 MB DOC) [file pone.0008895.s002.doc]

**Table S1.** Families, scientific names, common names, and exclusive trophic guild associations (derived from Randall 1967) of all fish species recorded from AGRRA sites used in this study.

| **FAMILY** | **SCIENTIFIC NAME** | **COMMON NAME** | **GUILD** |
| --- | --- | --- | --- |
| Acanthuridae | *Acanthurus bahianus* | Ocean surgeonfish | Herbivore |
| Acanthuridae | *Acanthurus chirurgus* | Doctorfish | Herbivore |
| Acanthuridae | *Acanthurus coeruleus* | Blue tang | Herbivore |
| Balistidae | *Balistes vetula* | Queen triggerfish | Invertebrate/bottom feeder |
| Balistidae | *Canthidermis sufflamen* | Ocean triggerfish | Coral/sponge/octocorallivore |
| Balistidae | *Melichthys niger* | Black durgon | Herbivore |
| Carangidae | *Caranx ruber* | Bar jack | Free-swimming predator |
| Chaetodontidae | *Chaetodon aculeatus* | Longsnout butterflyfish | Coral/sponge/octocorallivore |
| Chaetodontidae | *Chaetodon capistratus* | Foureye butterflyfish | Coral/sponge/octocorallivore |
| Chaetodontidae | *Chaetodon ocellatus* | Spotfin butterflyfish | Coral/sponge/octocorallivore |
| Chaetodontidae | *Chaetodon sedentarius* | Reef butterflyfish | Coral/sponge/octocorallivore |
| Chaetodontidae | *Chaetodon striatus* | Banded butterflyfish | Coral/sponge/octocorallivore |
| Haemulidae | *Anisotremus surinamensis* | Black margate | Invertebrate/bottom feeder |
| Haemulidae | *Anisotremus virginicus* | Porkfish | Invertebrate/bottom feeder |
| Haemulidae | *Haemulon album* | White margate | Invertebrate/bottom feeder |
| Haemulidae | *Haemulon aurolineatum* | Tomtate | Invertebrate/bottom feeder |
| Haemulidae | *Haemulon carbonarium* | Caesar grunt | Invertebrate/bottom feeder |
| Haemulidae | *Haemulon chrysargyreum* | Smallmouth grunt | Invertebrate/bottom feeder |
| Haemulidae | *Haemulon flavolineatum* | French grunt | Invertebrate/bottom feeder |
| Haemulidae | *Haemulon macrostomum* | Spanish grunt | Invertebrate/bottom feeder |
| Haemulidae | *Haemulon melanurum* | Cottonwick | Invertebrate/bottom feeder |
| Haemulidae | *Haemulon parra* | Sailors choice | Invertebrate/bottom feeder |
| Haemulidae | *Haemulon plumieri* | White grunt | Invertebrate/bottom feeder |
| Haemulidae | *Haemulon sciurus* | Bluestriped grunt | Invertebrate/bottom feeder |
| Labridae | *Bodianus rufus* | Spanish hogfish | Invertebrate/bottom feeder |
| Labridae | *Lachnolaimus maximus* | Hogfish | Invertebrate/bottom feeder |
| Lutjanidae | *Lutjanus analis* | Mutton snapper | Free-swimming predator |
| Lutjanidae | *Lutjanus apodus* | Schoolmaster | Free-swimming predator |
| Lutjanidae | *Lutjanus cyanopterus* | Cubera snapper | Free-swimming predator |
| Lutjanidae | *Lutjanus griseus* | Gray snapper | Free-swimming predator |
| Lutjanidae | *Lutjanus jocu* | Dog snapper | Free-swimming predator |
| Lutjanidae | *Lutjanus mahogoni* | Mahogany snapper | Free-swimming predator |
| Lutjanidae | *Lutjanus synagris* | Lane snapper | Free-swimming predator |
| Lutjanidae | *Ocyurus chrysurus* | Yellowtail snapper | Free-swimming predator |
| Monacanthidae | *Aluterus scriptus* | Scrawled filefish | Coral/sponge/octocorallivore |
| Monacanthidae | *Cantherhines macrocerus* | Whitespotted filefish | Coral/sponge/octocorallivore |
| Monacanthidae | *Cantherhines pullus* | Orangespotted filefish | Invertebrate/bottom feeder |
| Pomacanthidae | *Centropyge argi* | Cherubfish | Coral/sponge/octocorallivore |
| Pomacanthidae | *Holacanthus ciliaris* | Queen angelfish | Coral/sponge/octocorallivore |
| Pomacanthidae | *Holacanthus tricolor* | Rock beauty | Coral/sponge/octocorallivore |
| Pomacanthidae | *Pomacanthus arcuatus* | Gray angelfish | Coral/sponge/octocorallivore |
| Pomacanthidae | *Pomacanthus paru* | French angelfish | Coral/sponge/octocorallivore |
| Pomacentridae | *Microspathodon chrysurus* | Yellowtail damselfish | Herbivore |
| Scaridae | *Scarus coelestinus* | Midnight parrotfish | Herbivore |
| Scaridae | *Scarus coeruleus* | Blue parrotfish | Herbivore |
| Scaridae | *Scarus guacamaia* | Rainbow parrotfish | Herbivore |
| Scaridae | *Scarus inserti* | Striped parrotfish | Herbivore |
| Scaridae | *Scarus taeniopterus* | Princess parrotfish | Herbivore |
| Scaridae | *Scarus vetula* | Queen parrotfish | Herbivore |
| Scaridae | *Sparisoma atomarium* | Greenblotch parrotfish | Herbivore |
| Scaridae | *Sparisoma aurofrenatum* | Redband parrotfish | Herbivore |
| Scaridae | *Sparisoma chrysopterum* | Redtail parrotfish | Herbivore |
| Scaridae | *Sparisoma rubripinne* | Redfin parrotfish | Herbivore |
| Scaridae | *Sparisoma viride* | Stoplight parrotfish | Herbivore |
| Serranidae | *Cephalopholis cruentata* | Graysby | Sedentary predator |
| Serranidae | *Cephalopholis fulva* | Coney | Sedentary predator |
| Serranidae | *Epinephelus adscensionis* | Rock hind | Sedentary predator |
| Serranidae | *Epinephelus guttatus* | Red hind | Sedentary predator |
| Serranidae | *Epinephelus striatus* | Nassau grouper | Sedentary predator |
| Serranidae | *Mycteroperca bonaci* | Black grouper | Sedentary predator |
| Serranidae | *Mycteroperca interstitialis* | Yellowmouth grouper | Sedentary predator |
| Serranidae | *Mycteroperca tigris* | Tiger grouper | Sedentary predator |
| Serranidae | *Mycteroperca venenosa* | Yellowfin grouper | Sedentary predator |
| Sphyraenidae | *Sphyraena barracuda* | Great barracuda | Free-swimming predator |
