# Supplementary material for: Using Ecological Null Models to Assess the Potential for Marine Protected Area Networks to Protect Biodiversity
Source: PLoS One. 2010 Jan 27;5(1):e8895. doi: 10.1371/journal.pone.0008895 (PMC2811735; doi:10.1371/journal.pone.0008895)
Supplement: Table S2 — Habitat characteristics used to conduct the environmentally constrained null model co-occurrence tests, along with the methods used to quantify each characteristic for all sites. Citations given in the last column postulate, document through observation, or experimentally demonstrate associations (either positive or negative) between reef fish assemblage metrics (e.g., richness, recruitment, biomass, ordination) and the habitat characteristic. (0.12 MB DOC) [file pone.0008895.s003.doc]

Table S2. Habitat characteristics used to conduct the environmentally constrained null model co-occurrence tests, along with the methods used to quantify each characteristic for all sites. Citations given in the last column postulate, document through observation, or experimentally demonstrate associations (either positive or negative) between reef fish assemblage metrics (e.g., richness, recruitment, biomass, ordination) and the habitat characteristic.

| Habitat metric | Method used to derive metric | Sources |
| --- | --- | --- |
| Percent cover of macroalgae | Mean percent macroalgal cover from AGRRA benthic quadrats at each site. | [1,2,3,4] |
| Percent cover of crustose algae | Mean percent crustose algae cover from AGRRA benthic quadrats at each site. | [1,2] |
| Benthic complexity | Calculated product of mean coral diameter, mean coral height, and mean number of corals per AGRRA benthic transect at each site (method adapted from [5]). This calculation used both live and dead coral colonies recorded in transects; however only those corals over 25 cm diameter were used since a portion of transects only measured corals of this size or greater. | [6,7,8,9] |
| Percent cover of old dead coral | Mean percent cover of “old dead” coral (as defined by [5]) from AGRRA benthic quadrats at each site. | [10,11,12] |
| Percent cover of recently dead coral | Mean percent cover of “recently dead” coral (as defined by [5]) from AGRRA benthic quadrats at each site. | [10,11,13] |
| Percent cover of live coral | Mean percent live coral cover from AGRRA benthic quadrats at each site. | [13,14,15,16,17] |
| Percent cover of sand | Mean percent cover of sand from AGRRA benthic quadrats at each site. | [9,18,19,20,21] |
| Shannon (H’) diversity index of coral | H’ was calculated using the number and abundance of each coral species from AGRRA benthic transects at each site. | [14,22,23] |
| Connectivity/  habitat area | Circular buffers with a radius of 600 km were delimited around each site and a 500-m resolution grid of reef area [24] was used to calculate the area of reef habitat. | [25,26,27] |
| Latitude | Decimal degrees. | [9,28,29] |
| Longitude | Decimal degrees. | [9,28] |
| Index of fishing pressure | Circular buffers with a radius of 50 km were delimited around each site and a 500-m resolution grid of over-fishing threat levels from the Reefs at Risk database [24] was used to calculate the mean estimated over-fishing threat. | [30,31,32] |
| Depth | Estimated depth by averaging the depths of all AGRRA benthic transects conducted at each site. | [3,9,33] |
| Wave exposure | Exposure estimated by calculating maximum fetch to 1,000 km around each site. Fetch was calculated by drawing 1,000 km radiating lines from each site at 10 degree increments (i.e. 0, 10, … 350), and summing the total distance of these lines before crossing a shoreline [34]. | [8,21,35,36] |

REFERENCES

1. Levin PS (1993) Habitat Structure, Conspecific Presence and Spatial Variation in the Recruitment of a Temperate Reef Fish. Oecologia 94: 176-185.

2. McAfee ST, Morgan SG (1996) Resource use by five sympatric parrotfishes in the San Blas Archipelago, Panama. Marine Biology 125: 427-437.

3. Eagle JV, Jones GP, McCormick MI (2001) A multi-scale study of the relationships between habitat use and the distribution and abundance patterns of three coral reef angelfishes (Pomacanthidae). Marine Ecology-Progress Series 214: 253-265.

4. Tuya F, Boyra A, Sanchez-Jerez P, Barbera C, Haroun RJ (2004) Relationships between rocky-reef fish assemblages, the sea urchin Diadema antillarum and macroalgae throughout the Canarian Archipelago. Marine Ecology Progress Series 278: 157-169.

5. García-Charton JA, Pérez-Ruzafa A (1998) Correlation between habitat structure and a rocky reef fish assemblage in the southwest Mediterranean. PSZN Marine Ecology 19: 111-128.

6. Luckhurst BE, Luckhurst K (1978) Analysis of the influence of substrate variables on coral reef fish communities. Marine Biology 49: 317-323.

7. Gladfelter WB, Ogden JC, Gladfelter EH (1980) Similarity and Diversity among Coral-Reef Fish Communities - a Comparison between Tropical Western Atlantic (Virgin-Islands) and Tropical Central Pacific (Marshall-Islands) Patch Reefs. Ecology 61: 1156-1168.

8. Friedlander AM, Brown EK, Jokiel PL, Smith WR, Rodgers KS (2003) Effects of habitat, wave exposure, and marine protected area status on coral reef fish assemblages in the Hawaiian archipelago. Coral Reefs 22: 291-305.

9. Bouchon-Navaro Y, Bouchon C, Louis M, Legendre P (2005) Biogeographic patterns of coastal fish assemblages in the West Indies. Journal of Experimental Marine Biology and Ecology 315: 31-47.

10. Syms C, Jones GP (2000) Disturbance, habitat structure, and the dynamics of a coral-reef fish community. Ecology 81: 2714-2729.

11. Sano M (2000) Stability of reef fish assemblages: responses to coral recovery after catastrophic predation by Acanthaster planci. Marine Ecology Progress Series 198: 121-130.

12. Wilson S (2001) Multiscale habitat associations of detrivorous blennies (Blenniidae: Salariini). Coral Reefs 20: 245-251.

13. Lewis AR (1997) Effects of experimental coral disturbance on the structure of fish communities on large patch reefs. Marine Ecology Progress Series 161: 37-50.

14. Birkeland C, Neudecker S (1981) Foraging behavior of two Caribbean Chaetodontids: Chaetodon capistratus and C. aculeatus. Copeia 1981: 169-178.

15. Bell JD, Galzin R (1984) Influence of Live Coral Cover on Coral-Reef Fish Communities. Marine Ecology-Progress Series 15: 265-274.

16. Findley JS, Findley MT (1985) A Search for Pattern in Butterfly Fish Communities. American Naturalist 126: 800-816.

17. Jones GP, McCormick MI, Srinivasan M, Eagle JV (2004) Coral decline threatens fish biodiversity in marine reserves. Proceedings of the National Academy of Sciences of the United States of America 101: 8251-8253.

18. Jennings S, Boulle DP, Polunin NVC (1996) Habitat correlates of the distribution and biomass of Seychelles' reef fishes. Environmental Biology of Fishes 46: 15-25.

19. Booth DJ, Beretta GA (1994) Seasonal Recruitment, Habitat Associations and Survival of Pomacentrid Reef Fish in the United-States-Virgin-Islands. Coral Reefs 13: 81-89.

20. McCormick MI (1995) Fish Feeding on Mobile Benthic Invertebrates - Influence of Spatial Variability in Habitat Associations. Marine Biology 121: 627-637.

21. Depczynski M, Bellwood DR (2005) Wave energy and spatial variability in community structure of small cryptic coral reef fishes. Marine Ecology-Progress Series 303: 283-293.

22. Talbot FH (1965) A description of the coral structure of Tutia Reef (Tanganyika Territory, East Africa) and its fish fauna. Proceedings of the Zoological Society of London 145: 431-470.

23. Floeter SR, Guimaraes RZP, Rocha LA, Ferreira CEL, Rangel CA, et al. (2001) Geographic variation in reef-fish assemblages along the Brazilian coast. Global Ecology and Biogeography 10: 423-431.

24. Burke L, Maidens J (2005) Reefs at Risk in the Caribbean. Washington, D.C.: World Resources Institute. 80 p.

25. Cowen RK, Paris CB, Srinivasan A (2006) Scaling of connectivity in marine populations. Science 27: 522-527.

26. Bellwood DR, Hughes TP (2001) Regional-scale assembly rules and biodiversity of coral reefs. Science 292: 1532-1534.

27. Mora C, Robertson DR (2005) Factors shaping the range-size frequency distribution of the endemic fish fauna of the Tropical Eastern Pacific. Journal of Biogeography 32: 277-286.

28. Mora C, Robertson DR (2005) Causes of latitudinal gradients in species richness: A test with fishes of the Tropical Eastern Pacific. Ecology 86: 1771-1782.

29. Floeter SR, Ferreira CEL, Dominici-Arosemena A, Zalmon IR (2004) Latitudinal gradients in Atlantic reef fish communities: trophic structure and spatial use patterns. Journal of Fish Biology 64: 1680-1699.

30. Bell JD (1983) Effects of Depth and Marine Reserve Fishing Restrictions on the Structure of a Rocky Reef Fish Assemblage in the Northwestern Mediterranean-Sea. Journal of Applied Ecology 20: 357-369.

31. Wantiez L, Thollot P, Kulbicki M (1997) Effects of marine reserves on coral reef fish communities from five islands in New Caledonia. Coral Reefs 16: 215-224.

32. Halpern BS, Warner RR (2002) Marine reserves have rapid and lasting effects. Ecology Letters 5: 361-366.

33. Auster PJ, Semmens BX, Barber K (2005) Pattern in the co-occurrence of fishes inhabiting the coral reefs of Bonaire, Netherlands Antilles. Environmental Biology of Fishes 74: 187-194.

34. Ekebom J, Laihonen P, Suominen T (2002) Measuring fetch and estimating wave exposure in coastal areas. In: Gomes FV, Pinto FT, das Neves L, editors. Littoral 2002: 6th International Symposium Proceedings: a multi-disciplinary Symposium on Coastal Zone Research, Management and Planning, Porto, 22-26 September 2002. Porto, Portugal: EUROCOAST/EUCC.

35. Fulton CJ, Bellwood DR, Wainwright PC (2001) The relationship between swimming ability and habitat use in wrasses (Labridae). Marine Biology 139: 25-33.

36. Gust N, Choat JH, McCormick MI (2001) Spatial variability in reef fish distribution, abundance, size and biomass: a multi-scale analysis. Marine Ecology-Progress Series 214: 237-251.
